# Case Report Evidence of Relationships between Hepatocellular Carcinoma and Ochratoxicosis

**DOI:** 10.1371/journal.pone.0071423

**Published:** 2013-08-20

**Authors:** Ahmed S. Ibrahim, Hosam Zaghloul, Farid A. Badria

**Affiliations:** 1 Department of Biochemistry, Faculty of Pharmacy, Mansoura University, Mansoura, Egypt; 2 Department of Clinical Pathology, Faculty of Medicine, Mansoura University, Mansoura, Egypt; 3 Department of Pharmacognosy, Faculty of Pharmacy, Mansoura University, Mansoura, Egypt; University of Navarra School of Medicine and Center for Applied Medical Research (CIMA), Spain

## Abstract

**Purpose:**

The incidence of Hepatocellular carcinoma (HCC) is on the rise, but what is causing that trend has remained a mystery. Mycotoxins are almost entirely ignored health problems, and sometimes actually naively belittled in advanced medical care. Ochratoxin A (OTA) is one of the most abundant food contaminating mycotoxins worldwide that is carcinogenic, but no studies have evaluated its levels in HCC patients. Therefore, this study was designed to monitor the presence of OTA in the serum of HCC patients and to quantify the strength of the association between OTA and HCC.

**Methods:**

We conducted a case control-based study on 61 participants. Thirty-nine were HCC cases identified between 2010 and 2012 and individually matched by age, sex, residence and date of recruitment to 22 healthy controls. Serum OTA and alpha-fetoprotein levels were measured by using high-performance liquid chromatography (HPLC) and enzyme-linked immunosorbent assay, respectively.

**Results:**

HPLC analysis of 61 serum samples indicated that the highest incidence of elevated OTA was found in the HCC group and was 5-fold higher than in the control group. The concentration of OTA in the HCC group ranged between 0.129 and 10.93 ng/mL with a mean value±SD of 1.1±0.3 ng/mL, while in the normal group it ranged between 0.005 and 0.50 ng/mL with a mean value±SD of 0.201±0.02 ng/mL. The odds ratio for HCC patients presenting OTA levels above the cut-off of 0.207 (calculated by the receiver operating characteristic curve) was 9.78 (95% confidence interval = 2.9095–32.9816, *P* = 0.0002) with respect to normal controls, suggesting that HCC is 9.8 times as frequent in the exposed group to OTA.

**Conclusion:**

Our results reveal a strong association between the presence of OTA and HCC, which may offer a coherent explanation for much of the descriptive epidemiology of HCC and suggest new avenues for analytical research.

## Introduction

Hepatocellular carcinoma (HCC) is one of the most deadly malignancies worldwide [1]. Scientists have been studying the molecular mechanism of HCC for years, but the understanding of it remains incomplete and scattered across the literature at different molecular levels [2]. Chromosomal aberrations, epigenetic abnormality, and changes of gene expression have been reported as underlying mechanisms responsible for HCC development and progression [3]. However, which one of these mechanisms is predominant is vary among etiological factors and even vary among geographical regions [4]. Hepatitis B virus (HBV) and hepatitis C virus (HCV) infections and intake of alcohol are widely recognized as the major etiological factors of HCC [5]. Nonetheless, in 10–30% of HCC patients, no defined cause is found and therefore it seems likely that other risk factors also contribute to the development of HCC [6].

It is widely accepted that mycotoxins have adverse effects on human and animal health in many parts of the world [7]. Aflatoxins and ochratoxins are the most common mycotoxins contaminating a large fraction of developing countries' food, including maize, cereals, groundnuts and tree nuts [8]. Aflatoxin B has been extensively studied in relationship to liver cancer and has been well established as a hepatocarcinogenic in humans, particularly in conjunction with chronic hepatitis B virus infection [6], [9]. However, the contribution of ochratoxins to liver cancer is unknown and, because of its potential impact on the global disease burden, merits further clarification.

The ochratoxins are a group of mycotoxins produced by various Penicillium and Aspergillus species, with ochratoxin A (OTA) as the main analogue with considerable concern for human health. OTA proved to exhibit nephrotoxic, immunosuppressive, teratogenic and carcinogenic properties [10]. Several nephropathies affecting animals as well as humans have been attributed to OTA. This mycotoxin is the etiological agent of Danish porcine nephropathy and renal disorders observed in other animals. In humans, OTA is frequently cited as the possible causative agent of Balkan endemic nephropathy, a syndrome characterized by contracted kidneys with tubular degeneration, interstitial fibrosis and hyalinization of the glomeruli [11]. Despite occasional high profile incidents, such as acute poisoning outbreaks or the presence of mycotoxins in nutritional supplements, OTA has not been widely prioritized from a public health perspective in low income countries [8]. The Scientific Committee for Food has concluded that the intake of OTA should be reduced as far as possible, i.e. below 5 ng/kg body weight/day [12]. However, OTA is widely detected in cereals and cereal-derived products, wheat, barley, rice and sorghum [13], [14]. Additionally, OTA is found in high amounts in animal foodstuffs [15]. Where these commodities are dietary staples, the contamination translates to high level chronic exposure [8].

In 1993, the International Agency for Research on Cancer classified OTA in group 2B, a possible human carcinogen based on sufficient evidence of carcinogenicity from studies in experimental animals [16]. Oral exposure to ochratoxins caused tumors at several different tissue sites in mice and rats. Dietary administration of ochratoxins caused benign and/or malignant liver tumors in mice of both sexes and benign and malignant kidney tumors in male mice [17], [18]. The presence of OTA in human sera, due to the ingestion of several contaminated foods such as cereals [19], [20], is related to its slow elimination, resulting in having a half-life of about 35 days in serum [21]. The data available from epidemiological studies are inadequate to evaluate the relationship between human liver cancer and exposure specifically to OTA. Therefore, this study was designed to monitor the presence of OTA in the serum of HCC patients and to quantify the strength of the association between OTA and HCC.

## Materials and Methods

### Population for analysis and sampling

The current investigation is a case-control based study, which was approved by the ethical institutional review board at Mansoura University that complies with acceptable international standards. Written informed consent for participation was obtained from each participant. Case patients were recruited from the population of patients with diagnosed HCC who were evaluated and treated at Mansoura Cancer Center. The inclusion criteria were as follows: pathologically confirmed diagnosis of HCC, α-fetoprotein (AFP) >20 ng/mL, a diagnostic cut-off value, and Egyptian residency. The exclusion criteria were the presence of other types of primary liver cancer (such as cholangiocarcinoma or fibrolamellar hepatocarcinoma), unknown primary tumors, AFP <20 ng/mL and concurrent or past history of cancer at another organ site.

From January 2010 through August 2012, 50 patients with suspected HCC were identified, 42 of whom were eligible for this study. We enrolled 39 eligible patients with HCC; 3 eligible patients were not used because of patient refusal or limited blood samples. Statistical analyses indicated that the eligible patients who were not recruited did not differ from the recruited patients in terms of demographical, epidemiological or clinical factors (retrieved from patients' medical records).

The control subjects were healthy and recruited from the diagnostic biochemical lab, AutoLab of Mansoura institution, and were matched by age, sex and ethnicity to the case subjects. The eligibility criteria for controls were the same as those for patients, except for having a cancer diagnosis. A short structured questionnaire was used to screen for potential controls on the basis of the eligibility criteria. Analysis of the answers received on the short questionnaire indicated that 80% of those questioned agreed to participate in clinical research. A total of 29 eligible control subjects were ascertained in the current study. However, 7 control subjects were excluded due to limited blood samples for testing AFP and OTA. Blood samples (5 mL) were taken, centrifuged and the serum separated and stored at −20°C until analyzed. Serum samples were assayed for AFP by enzyme-linked immunosorbent assay with commercial kits (Abbott, North Chicago, IL), for OTA by high performance liquid chromatography (HPLC), and for liver function markers, alanine aminotransferase (ALT) and aspartate aminotransferase (AST), with an auto-analyzer (Hitachi Model 736, Japan) and commercial kits (BioMerieux, United States).

### Chemicals and reagents

Chemicals and solvents were of HPLC grade or equivalent. All water used was deionized and, for HPLC, obtained from a Milli-Q purification system (Millipore, London, United Kingdom). Acetonitrile, methanol, sodium acetate and acetic acid used for mobile phases were of HPLC grade and provided by Fisher Scientifics (Fisher chemicals HPLC, United States). The column for OTA was purchased from Vicam (Milford, MA, United States). All the analyses were performed in subdued light. OTA standard was provided by Sigma Chemicals (St. Louis, MO, United States). It was dissolved in methanol. A stock solution of OTA was prepared by solving 5 mg of OTA in 1 mL of methanol (HPLC grade). Standard curve solutions were prepared from appropriate dilutions of the stock solution with methanol.

### Determination of ochratoxin A

The OTA concentration was determined in the serum of HCC patients as well as controls by HPLC and microfluorometric detection by the same method used by Wafa *et al* [22], with some modification. Briefly, to 100 μL of serum, 400 μL of absolute methanol (HPLC grade) was added, mixed vigorously and allowed to stand overnight at 4°C. Then the sample was centrifuged and the supernatant was clarified by filtration. 20 μL of clarified supernatant was injected directly into HPLC.

### Liquid chromatography conditions

Mobile phase: methanol, acetonitrile, sodium acetate 5 mm, acetic acid (300: 300: 400: 14 v/v/v/v); injection volume: 20 µL; flow rate: 1.5 mL/minute; fluorescent detection: excitation at 340 nm and emission at 465 nm. The quantification of OTA was achieved automatically by the computer, according to the peak high of three OTA standards (0.5 ng/mL, 5 ng/mL and 20 ng/mL) injected sequentially with ochratoxin-free methanol.

### Statistical analysis

All analyses were performed with the GraphPad Prism 3 package. Data were checked for skewness and an unpaired *t*-test was performed if the distribution of the values was Gaussian. If the distribution was not normal, a non-parametric test, Mann-Whitney test, was used. P values less than 0.05 were considered to be statistically significant. Receiver operating characteristic (ROC) curve analysis was performed with MedCalc statistical software for biomedical research. Odds ratios were determined by χ2 test (GraphPad Prism 5).

## Results

### Validation of the analytical method used

**Figure 1:**
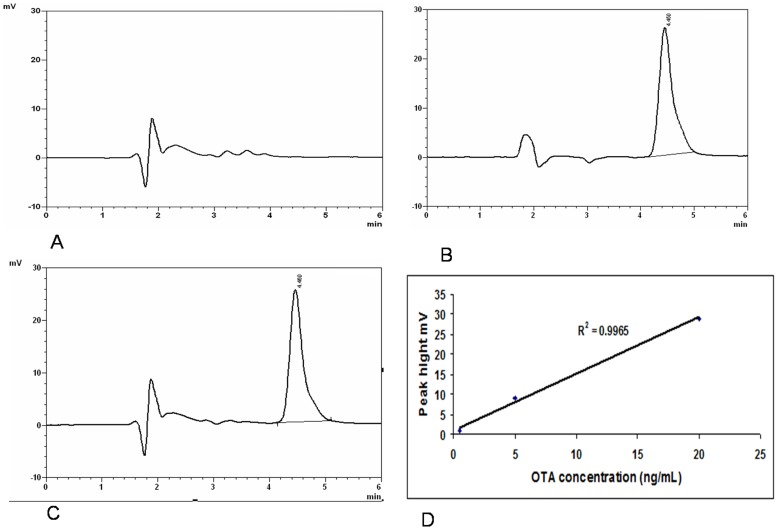
Validation of the analytical method. A: HPLC chromatograms of OTA-free serum; B: OTA standard solution 20 ng/mL; C: 20 ng/mL OTA-spiked serum, OTA retention time was 4.2±0.3 min; D: Dose–response curve for OTA spiked in serum. Plotted experimental points are the average of three determinations. HPLC: High-performance liquid chromatography; OTA: Ochratoxin A.

In order to evaluate OTA contamination levels in human sera, we successfully validated a chromatographic analytical method by the adaptation of the method given by Wafa *et al* [22] with some modifications. The validation of this method was based on the criteria of linearity, reproducibility, repeatability and recovery. Under the chromatographic conditions used, the retention time of OTA was found to be about 4.2±0.3 minutes, Figure 1A and B. Recovery experiments were determined by spiking OTA-free samples of human serum with OTA at the level of 20 ng/mL in the same day, by the same operator and with the same HPLC system. The average recovery using the modified procedure was: 97%±3%, Figure 1C. The calibration curve, using different concentrations, was generated by plotting OTA-spiked serum peak highs against the corresponding concentrations of calibration samples (0.5 ng/mL, 5 ng/mL and 20 ng/mL), Figure 1D. All objectives for linearity validation were matched; coefficient of correlation *R*2  = 0.9965, indicating a good calibration curve, Figure 1D.

### Characteristics of patients

The characteristics of the patients are summarized in Table 1. In brief, most of the HCC patients were males (M/F  = 33/6) and their ages ranged between 40–70 years with a mean ± SD of 55.9±6.7 years. Moreover, as shown in Table 1, there were no significant differences with respect to age and sex between patients and controls. It is well documented that AFP estimation remains a useful test for oncologists involved in the management of patients with HCC. AFP has been used for many years as a convenient biomarker for HCC because of its associations with liver carcinogenesis and was recently recognized as linked with aggressive tumor behavior [23]. Accordingly, AFP analysis was carried out in all patients. Laboratory investigations (Table 1) showed a markedly elevated serum AFP concentration in the HCC group (4120 ng/mL), together with deranged liver function tests (ALT: 26.7 U/mL, AST: 59.67 U/mL, AST/ALT: 2.37, albumin: 3.13 g/dL). The highest abnormal value of AFP detected was 18800 ng/mL and the elevated values are in the range of 132–18800 ng/mL. The normal cut-off value for adults is less than 20 ng/mL [24].

**Table 1:** Clinical characteristics of hepatocellular carcinoma patients and controls (mean ± SD).

Parameter	Hepatocellular carcinoma patients (*n* = 39)	Healthy controls (*n* = 22)
Age	55.9±6.7	53.4±5.8
Sex (Male:Female)	33:6	19:3
AST (U/mL)	59.67±13.05^1^	12.7±1.4
ALT (U/mL)	26.7±6.34^1^	16.8±1.9
AST/ALT	2.37±0.89^1^	0.63±0.05
Albumin (g/dL)	3.13±0.152^1^	4.71±0.17
AFP (ng/mL)	4120±5520^1^	<20 (5.3±0.87)

1 Significantly different from the controls at *P*<0.001. ALT: Alanine aminotransferase; AST: Aspartate aminotransferase; AFP: Alpha-fetoprotein.

### Serum OTA levels in healthy subjects and in HCC patients

**Figure 2:**
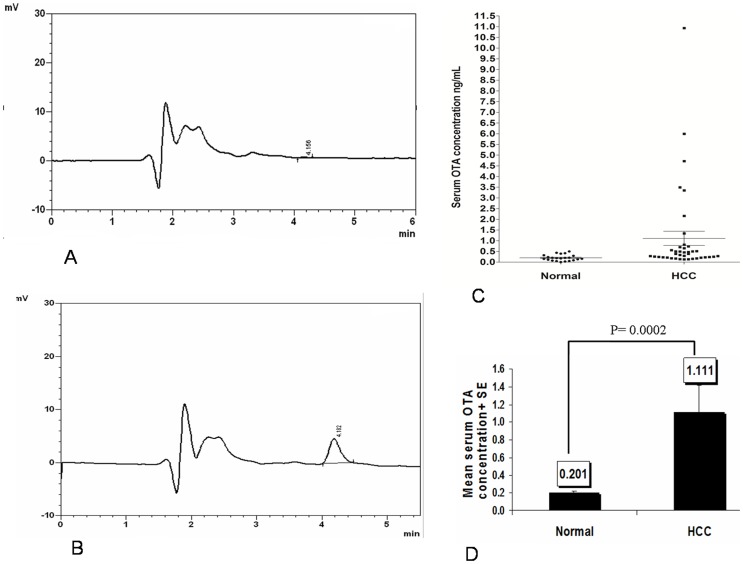
Serum ochratoxin A levels in healthy subjects and in hepatocellular carcinoma patients. A: HPLC chromatograms for OTA in a representative control; B: HPLC chromatograms for OTA in a representative HCC serum; C: Dispersion diagram for OTA concentration in human sera from healthy and HCC groups; D: Statistical analysis of OTA levels in HCC and normal groups. HPLC: High-performance liquid chromatography; OTA: Ochratoxin A; HCC: Hepatocellular carcinoma.

To explore the association between OTA and HCC, we first measured the serum levels of OTA in HCC patients and healthy subjects. HPLC analysis of 61 serum samples indicated that the highest incidence of elevated OTA was found in the HCC group and it was 5-fold higher than in the control group. HPLC chromatograms of OTA-free serum and OTA naturally contaminated serum are represented in Figure 2A and B. The concentration of OTA in the HCC group ranged between 0.129 and 10.93 ng/mL with a mean value ± SE of 1.11±0.3 ng/mL, while in normal group, it ranged between 0.005 and 0.50 ng/mL with a mean value ± SE 0.201±0.02 ng/mL, Figure 2C. Statistical analysis showed a significant difference in OTA levels between these groups (*P* = 0.0002), Figure 2D.

**Figure 3:**
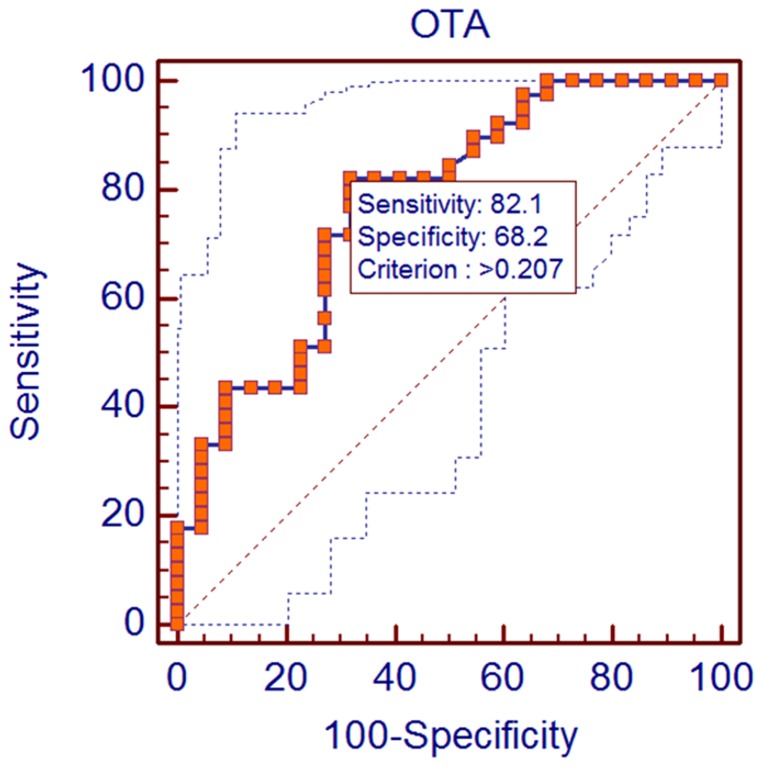
Receiver operating characteristic curve of optimal cut-off value dichotomizing patients with high or low ochratoxin A exposure. OTA: Ochratoxin A.

After having shown that the OTA level was increased significantly among HCC patients, we next sought to quantify the strength of the association between the presence of OTA and HCC. Therefore, we used the odds ratio to contrast the odds of exposure among cases with the odds of exposure among controls. In this regard, the samples were dichotomized as high or low OTA exposure based on detection of OTA above the cut-off value. This cut-off limit was determined by using ROC curves analyzing the levels and distribution of OTA in normal and diseased samples. The ROC curve obtained by plot at different cut-offs is shown in Figure 3. According to ROC analyses, the area under the curve was AUC  = 0.772 with SE  = 0.0644, *P*<0.0001 and 95% CI from 0.647 to 0.870. Moreover, the statistical software found that the optimum cut off criterion that maximizes sensitivity and specificity in screening for OTA in HCC patients was 0.207 ng/mL, from a large number of detection criteria, Figure 3. At this concentration, the sensitivity was 82.1% and specificity 68.2%, respectively.

Based on this classification, the odds ratio for an association between the presence of OTA and HCC was calculated. It demonstrated that (OR) for HCC patients presenting OTA levels above the cut-off of 0.207 (calculated by the ROC curves) was 9.8 (95% confidence interval  = 2.9095–32.9816, *P* = 0.0006) with respect to normal controls, Table 2, suggesting that HCC is 9.8 times as frequent in the exposed group to OTA. Moreover, the patients' group exhibited significantly (*P*<0.01) higher rates of OTA-positive samples than healthy subjects (82% *vs* 32%, *P*<0.001), Table 2.

**Table 2:** Rate of ochratoxin A-positive samples and risk of hepatocellular carcinoma in patients presenting ochratoxin A levels above or below the cut-off of 0.207 (calculated by the receiver operating characteristic curves) compared with controls.

OTA exposure index	HCC patients		Controls		OR^1^	95% Cl^2^
	N	%	N	%		
**High**	32	**82** 3	7	**32**	9.795918	2.9095 to 32.9816
**Low**	7	**16**	15	**68**		

1 OR relative to an increase of 1 standard deviation among controls.

2 *P* = 0.0006.

3 *P*<0.001 Compared to the control group.

OR: Odd ratio.

CI: Confidence interval; OTA: Ochratoxin A.

## Discussion

Hepatocellular carcinoma (HCC) is now regarded as one of the major malignant diseases worldwide, with significant variations in its epidemiology. HCC is unusual among human cancers in that its pathophysiology is complex, multifactorial and ever-expanding. There are multiple etiological factors affecting HCC, all of which vary by geographical location and have a direct impact on the characteristics of these patients, making HCC an extremely complex condition [25], [26]. HCV and HBV infections and alcohol drinking are major culprit risk factors recognized for HCC, responsible for approximately 80% of new cases [4], [27], [28]. Nonetheless, in 10–30% of HCC patients, no defined cause is found. Therefore, it is conceivable that risk factors other than viral infection or alcohol drinking exist for HCC, but the evidence is still unclear. Mycotoxins are almost entirely ignored health problems and sometimes actually naively belittled in advanced medical care. In this study, we put forward a new concept pertaining to the possible association of ochratoxin exposure with HCC risk. Ochratoxins are important food-borne health threats [29], [30]. The co-contamination of foodstuffs with these toxins is well known in developing countries, such as Egypt [31], and has been implicated in a wide numbers of acute and chronic human diseases, including nephropathy [22].

OTA is an ubiquitous ochratoxin in human blood, exhibiting unusual toxicokinetics with a half-life of 840 hours (35 days), thus providing unquestionable information on the quantification of ochratoxicosis in body fluids and being an indicator of continuous exposure to such toxins [32]. Therefore, in the current work, risk assessment of ochratoxicosis in HCC was evaluated with the help of this biomarker, which provides data on such exposure and evaluates the possible human health risk of ochratoxins. OTA elimination occurs primarily via the kidney and liver, either as metabolites or unchanged. Two metabolic products were formed from OTA by human liver microsomal fractions in the presence of reduced nicotinamide adenine dinucleotide phosphate, 4 (R)- and 4 (S)-OH-OTA, with the 4R isomer being the major one [33]. It had been speculated that the toxic effects of OTA could be reduced by the formation of its metabolic isomers during long-term exposure of small amounts of the toxin because (4R)-OH-OTA appeared to be nontoxic when given to rats at a dose of 40 mg/kg [34]. Furthermore, liver elimination of OTA is additionally maintained by protein carriers that shuffle the toxin from its protein-bound form in blood into the hepatocyte and subsequently secrete the toxin into bile. The uptake carrier has been identified but less is known about the mechanism involved in the release of the toxin into bile. A carrier system is also involved in the uptake of OTA by proximal tubule cells, which secrete the toxin into urine. The delayed excretion of the toxin may be attributed to the re-absorption during enterohepatic circulation, decreased metabolism of OTA, re-absorption from the urine after tubular secretion, or extensive protein binding. Such systems are biological entrance gates that determine the elimination toxicokinetics of OTA and therefore have a major impact on the half-life time and the selective organ exposure [35].

Epidemiological studies have provided evidence for a correlation between high OTA levels in blood and food samples and the incidence of human nephropathies [36]. Furthermore, OTA seems to be implicated in the pathogenesis of Balkan endemic nephropathy (BEN) [37], [38]. However, there is no proof of cause and effect, but the close association between the BEN and OTA is far too great to be coincidental [39]. OTA is not only a specific nephrotoxin but it also impairs different cellular functions, giving rise to metabolic disorders and immunosuppressive and teratogenic effects [40]. In humans, exposure to high levels of OTA in the diet has been linked with an increased incidence of urinary tract tumors [39]. In experimental animals, OTA induces tumors in the kidney as well as in the liver [41], [42]. Although several lines of evidence derived from animal experiments implicate OTA in hepatic carcinogenesis [16], [17], [18], no data is available from epidemiological studies to evaluate such a relationship. Hence, the current study is the first to report the presence of OTA in the serum of HCC patients in a ratio approximately 5 times higher than in controls. This difference in exposure continued to show a possible link between OTA exposure and HCC. Furthermore, since OTA is eliminated by the liver, hepatic diseases may prolong its serum half-life and the possibility that the observed difference in OTA exposure is due to diminished excretion in HCC patients cannot be ruled out.

Detection limits for OTA using liquid chromatographic methods are about 0.005–0.1 ng/mL plasma/serum so that incidences of positives often are 50–100%, reflecting widespread and continuous exposure of humans to OTA [32]. The presence of OTA in healthy population sera with a low concentration, which is in agreement with several other studies in more than 20 countries [32], without developing HCC supports the involvement of genetic predisposition in human hepatoma [43]. The hypothesis of a genetic abnormality or differential polymorphism of metabolizing enzymes of OTA can be evoked [44].

The presence of OTA in human sera was shown to be related to OTA-contaminated food ingestion [19], [20]. Several studies have shown a regular and high incidence of OTA contamination in a large number of frequently consumed foods, such as cereals, cereal-related products, beans, rice germ, rice germ cake, broilers feed, egg production feed and milk production feed in Egypt [45], [46]. The observed high incidence and levels of OTA contamination in Egyptian foods could be explained, firstly, by the climatic conditions, especially humidity and temperature which are in favor of toxigenic fungi proliferation [47], and secondly, by the social and economic characteristics of the Egyptian population, such as the cooking methods, food storage and eating habits [46].

Another important point of this work is the demonstration of a strong association between OTA and HCC, as indicated by a high odds ratio, showing that HCC is 9.8 times as frequent in the exposed group to OTA. This evaluation is essential with the aim to determine the contribution of OTA exposure to HCC pathogenesis, suggesting that OTA is a plausible etiological factor in HCC. This input has originated partly from histopathological studies that show neoplastic nodules and tumors in the liver of male ddY and DDD mice treated with OTA [42]. These initial observations have been supported in B6C3F1 mice [42] and reinforced by additional studies showing that OTA causes liver DNA damage and cytogenetic effects in rats [48], [49]. Moreover, the *in vitro* studies on rat liver epithelial cells treated with OTA have shown the tumor promoting properties of OTA [50]. All of this evidence has sculpted the concept of OTA as a contributing factor in heptocarcinogenesis.

Accordingly, the direct toxic effect of OTA on hepatocytes has been explored through *in vitro* studies. HCC is a multistage process enumerated by initiation, promotion and progression steps and it implicates that identification of the step where OTA interferes with this process would be of significance in understanding the risk associated with its exposure. Both non-genotoxic and genotoxic mechanisms have been reported to be the underlying contributors to tumor formation by OTA. Previously, Horvath *et al* [50] reported that the nongenotoxic mechanisms of OTA to induce hepatocarcinogenecity were mediated via its ability to inhibit gap junctional intercellular communication (GJIC), a cellular mechanism of tumor promotion. However, the precise mechanism by which OTA inhibits GJIC is not fully understood. On the other hand, it has been well established that DNA damage is an important event in initiating carcinogenesis [51]. Although there is no evidence of OTA-DNA adduct formation as metabolism of OTA fails to generate electrophilic intermediates [52], DNA damaging potential of OTA has been reported by various investigators both *in vivo* and *in vitro* [48], [49], [53]. Therefore, it has been suggested that reactive oxygen species (ROS) and oxidative DNA damage could be one of the causative factors in OTA induced toxicity and tumorigenicity [54].

The major group of signaling molecules which are modulated by ROS is mitogen activated protein kinases (MAPKs) [55]. MAPKs are a superfamily of serine/threonine kinases which are activated by many exogenous or endogenous stimuli and play a pivotal role in cell survival and death. There are three major MAPKs identified in mammalian cells in which extracellular receptor kinase (ERK1/2) and p38 are central to mitogenesis [56]. As such, OTA has been reported to promote tumor formation in liver cells by activating ERK and p38-mediated cell proliferation. Exposure to OTA led to the activation of both ERK1 and ERK2 in rat liver cells up to 24 hours. Activation began at 4 hours and was concentration dependent. Furthermore, using a phospho-specific antibody to the activated form of p38 MAPK, a transient activation was observed peaking at 16 hours of incubation with OTA [50]. Collectively, these GJIC- and MAPK-results suggest a mechanism for the high hepatic cancer rate observed after 30 weeks of treatment of rodents with 40 mg/kg OTA [57].

Taken together, although this study is limited by its retrospective nature, it demonstrates that the chronic exposure to a high level of OTA could increase the risk of liver cancer. Future epidemiological studies of HCC should focus prospectively on feeding practices and the consumption of OTA-containing foods, such as cereals and milk, by liver-diseased patients. Since exposure to OTA is common, its effects on the development of HCC make it an important public health concern. Therefore, an active food surveillance program to reduce or eliminate OTA exposure is warranted.
